# Case Report: A Novel Homozygous Missense Variant of *FBN3* Supporting It Is a New Candidate Gene Causative of a Bardet–Biedl Syndrome–Like Phenotype

**DOI:** 10.3389/fgene.2022.924362

**Published:** 2022-07-15

**Authors:** Maria Luce Genovesi, Barbara Torres, Marina Goldoni, Eliana Salvo, Claudia Cesario, Massimo Majolo, Tommaso Mazza, Carmelo Piscopo, Laura Bernardini

**Affiliations:** ^1^ Department of Experimental Medicine, Sapienza University of Rome, Rome, Italy; ^2^ Medical Genetics Division, IRCCS Casa Sollievo Della Sofferenza Foundation, San Giovanni Rotondo, Italy; ^3^ Medical Genetics, Department of Biomedical and Biotechnological Sciences, University of Catania, Catania, Italy; ^4^ Translational Cytogenomics Research Unit, Bambino Gesù Children’s Hospital, IRCCS, Rome, Italy; ^5^ Hospital Directorate, National Hospital A.O.R.N. “Antonio Cardarelli”, Naples, Italy; ^6^ Laboratory of Bioinformatics, IRCCs Casa Sollievo Della Sofferenza Foundation, San Giovanni Rotondo, Italy; ^7^ Medical and Laboratory Genetics Unit, National Hospital A.O.R.N. “Antonio Cardarelli”, Naples, Italy

**Keywords:** Bardet–Biedl syndrome, *FBN3*, fibrillinopathies, SNP-array, whole-exome sequencing

## Abstract

Fibrillin proteins are extracellular matrix glycoproteins assembling into microfibrils. *FBN1*, *FBN2*, and *FBN3* encode the human fibrillins and mutations in *FBN1* and *FBN2* cause connective tissue disorders called fibrillinopathies, affecting cardiovascular, dermal, skeletal, and ocular tissues. Recently, mutations of the less characterized fibrillin family member, *FBN3*, have been associated in a single family with Bardet–Biedl syndrome (BBS). Here, we report on a patient born from two first cousins and affected by developmental delay, cognitive impairment, obesity, dental and genital anomalies, and brachydactyly/syndactyly. His phenotype was very similar to that reported in the previous *FBN3*-mutated family and fulfilled BBS clinical diagnostic criteria, although lacking polydactyly, the most recurrent clinical feature, as the previous siblings described. A familial SNP-array and proband’s WES were performed prioritizing candidate variants on the sole patient’s runs of homozygosity. This analysis disclosed a novel homozygous missense variant in *FBN3* (NM_032447:c.5434A>G; NP_115823:p.Ile1812Val; rs115948457), inherited from the heterozygous parents. This study further supports that *FBN3* is a candidate gene for a BBS-like syndrome characterized by developmental delay, cognitive impairment, obesity, dental, genital, and skeletal anomalies. Anyway, additional studies are necessary to investigate the exact role of the gene and possible interactions between FBN3 and BBS proteins.

## Introduction

Fibrillin proteins are extracellular matrix glycoproteins that assemble into microfibrils with architectural functions in many connective tissues (Corson et al., 2004; Sabatier et al., 2011). These 10–12 nm extracellular calcium-binding microfibrils occur either in association with elastin or in elastin-free bundles and provide long-term force bearing structural support (Corson et al., 2004). Fibrillins and microfibrils are also involved in matrix deposition and activation of growth factors belonging to the transforming growth factor β (TGF-β) superfamily, including TGF-β and bone morphogenetic proteins (BMPs), which regulate a broad array of developmental and homeostatic processes (Reinhardt, 2014).

*FBN1* (*fibrillin 1*; MIM * 134797), *FBN2* (*fibrillin 2*; MIM * 612570), and *FBN3* (*fibrillin 3*; MIM * 608529) encode the three human fibrillins that show a highly conserved modular domain organization and about 61–69% of homology at the amino acid level and are also conserved among species (Hubmacher et al., 2006).

*FBN1* is expressed throughout life, whereas *FBN2* and *FBN3* are primarily expressed during embryonic development. Indeed, the developing embryo seems to require all the three fibrillins, while the adult organism only fibrillin-1 (Corson et al., 2004; Sabatier et al., 2011). Mutations in *FBN1* and *FBN2* cause connective tissue disorders called fibrillinopathies. *FBN1* has been associated with several autosomal dominant (AD) disorders: acromicric dysplasia (MIM # 102370), ectopia lentis familial (MIM # 129600), geleophysic dysplasia 2 (MIM # 614185), Marfan lipodystrophy syndrome (MIM # 616914), Marfan syndrome (MIM # 154700), MASS syndrome (MIM # 604308), stiff skin syndrome (MIM # 184900), and Weill–Marchesani syndrome 2 (MIM # 608328). *FBN2* is causative of AD congenital contractural arachnodactyly (MIM # 121050) and AD macular degeneration early-onset (MIM # 616118). These fibrillinopathies share some common features and are all characterized by clinical manifestations in cardiovascular, dermal, skeletal, and ocular tissues. Sometimes also palate malformations and neurologic features are reported (Robinson et al., 2006). *FBN3* seems to play a role in the pathogenesis of polycystic ovary syndrome 1 (PCOS1; MIM % 184700) (Urbanek et al., 2005; Stewart et al., 2006; Ewens et al., 2010; Jordan et al., 2010). Recently, the homozygous missense variant of *FBN3* (NM_032447) c.2804G>A (p.Arg935Gln) has been associated with Klippel–Trenaunay–Weber syndrome (MIM % 149000) (Liu et al., 2019), while the compound heterozygous variants c.3616G>A (p.Val1206Ile) and c.6037C>T (p.Arg2013Trp) have been associated with Bardet–Biedl syndrome 1 (BBS1; MIM # 209900) (Wang et al., 2017), a multisystemic ciliopathy characterized by retinal dystrophy, obesity, post-axial polydactyly, renal dysfunction, learning difficulties, and hypogonadism (Forsythe and Beales, 2013; Niederlova et al., 2019; Forsyth and Gunay-Aygun, 2003).

In this work, we report on a patient with a homozygous *FBN3* missense variant identified by a combined SNP-array and whole-exome sequencing approach and a phenotype characterized by cognitive impairment, developmental delay, learning difficulties, strabismus, arched palate, dental and genital anomalies, brachydactyly/syndactyly, and obesity, supporting that *FBN3* alteration could be associated with a phenotype resembling BBS.

## Materials and Methods

### Case Report

**FIGURE 1:**
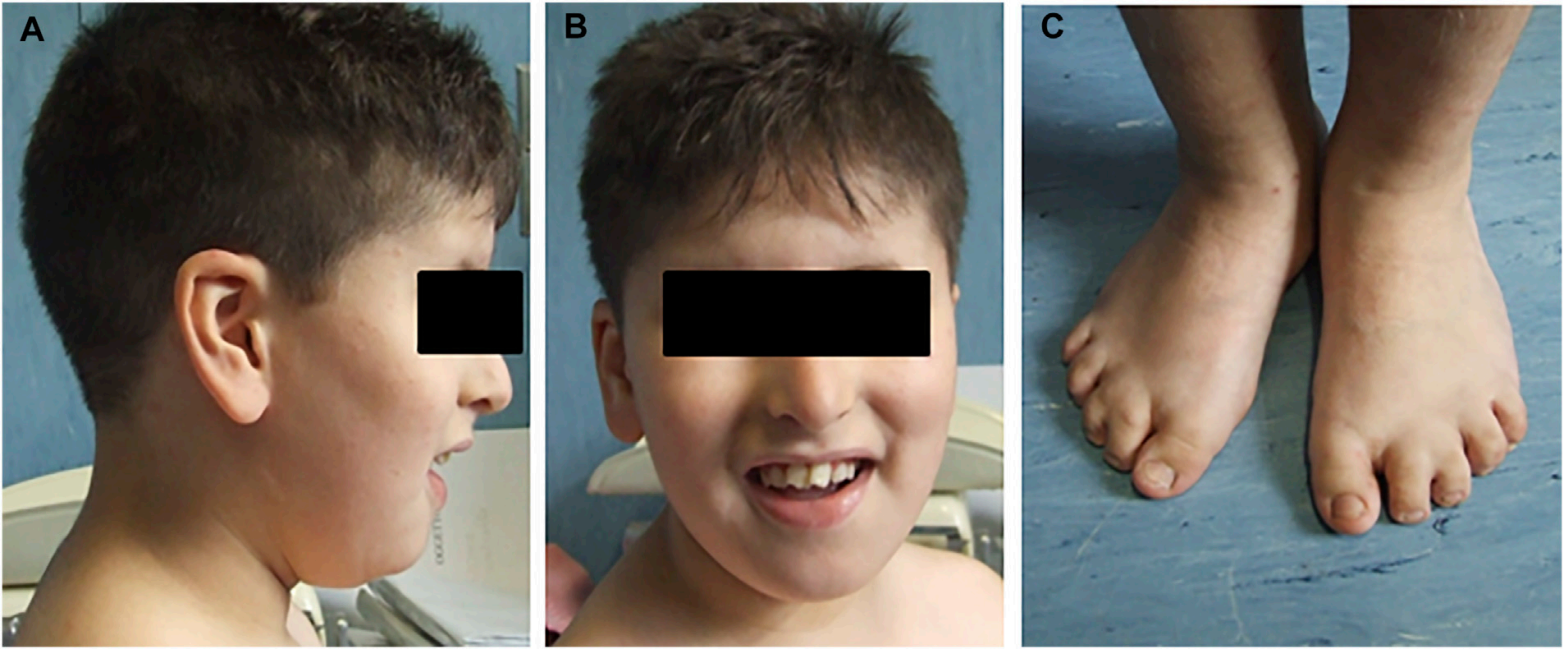
Pictures report some clinical characteristics of the proband. **(A)** Facial dysmorphisms, beaked nose, and large ears. **(B)** Dental abnormalities. **(C)** Bilateral brachydactyly and syndactyly between II and III toes fingers.

Second of four siblings, the proband was born from consanguineous non-affected parents (first cousins) of Moroccan origin at full term (39W) of a physiological pregnancy and after natural delivery, with a birth weight of 2,500 kg (5–10 percentile). He was evaluated at the age of 8 years for delayed psychomotor development, learning difficulties, impaired verbal fluency and attention capacity, speech delay, and gross and fine motor abnormalities. Clinical examination revealed various facial dysmorphisms: hypotelorism, eyelid fissures tilted down, beaked nose, arched palate, and large ears. He also showed microdontia with periodontal disease and numerous dental caries, with the removal of three teeth. He had a mild strabismus with hyperopia, bilateral brachydactyly and syndactyly between II and III toes fingers, micropenis with right cryptorchidism, and central obesity (Figure 1). He suffered from frequent respiratory infections and mild obstructive apnea. Audiological tests revealed bilateral conductive hearing loss. The last ophthalmological examination, performed at age 9, documented optic discs with fuzzy, hyperemic margins, and marked vascular tortuosity. He regularly followed a rehabilitation therapy for several years. No abnormalities were found after cardiological, gastroenterological, and neurological investigations, including brain MRI and E.E.G. At the last evaluation (age 11), he had normal head circumference and stature parameters (respectively 51.5 and 137 cm) but a significant overweight, with +2DS of both weight and BMI (respectively, 52 kg and 27.7). FRAXA and methylation for Prader–Willi syndrome were normal.

### SNP-Array

Genomic screening for copy number variations (CNVs) was performed on the proband and, successively, on his three healthy siblings’ DNA using a SNP-array platform (GeneChip 6.0, Affymetrix, Santa Clara, CA, United States), carried out following the manufacturer’s recommendations and analyzed using ChAS software (v3.1; Affymetrix). A total of 270 healthy controls belonging to the International HapMap Project were used as a reference sample in data analysis (Affymetrix). Called CNVs were represented by at least 25 contiguous probes and 75 kb as a minimum size and were classified according to the American College of Medical Genetics (ACMG) recommendations (Riggs et al., 2020). Moreover, all CNVs represented by at least five contiguous probes and laying within a disease-gene (OMIM, Online Mendelian Inheritance in Man; https://www.omim.org/) were considered. Runs of homozygosity (ROH) analysis of autosome chromosomes was performed using the SNPs and filtered considering 3 Mb of length as a minimum size (Kearney et al., 2011).

### Whole-Exome Sequencing (WES)

WES on the proband’s genomic DNA was performed at CRIBI (Centro di Ricerca Interdipartimentale per le Biotecnologie Innovative) Genomics (Padua, Italy), using the Ion TargetSeq™ Exome Enrichment Kit (Applied Biosystems, Foster City, CA, United States) as an exome capture kit and the Ion Proton™ System (Applied Biosystems) as a sequencing platform. Reads alignment to the human genome (UCSC GRCh37/hg19) and variant calling were performed through the Torrent Suite Software v5.2.1. Variants and gene annotation was performed using ANNOVAR (Wang et al., 2010; July 2017 release) and resorting to the dbNSFP v3.5 gathered annotations (Liu et al., 2016). Single nucleotide variants (SNVs) and small insertions and deletions (Indels) were filtered based on quality criteria and their effect, only high-quality variants and those with an effect on the coding sequence and splice site regions (±10 bp) were retained. The resulting variants were further filtered to retain only those with unknown frequency or a minor allele frequency (MAF) ≤1%, according to the gnomAD database (v2.1.1), and prioritized based on their predicted functional impact, evaluated through the Combined Annotation Dependent Depletion (CADD) score (v1.6), using a value of 10 as a threshold (Kircher et al., 2014). Subsequently, the variants were first prioritized on the basis of SNP-array results, considering all the ROH identified in the patient and not shared with his healthy siblings, and then considering different modes of inheritance, in order to exclude damaging variants in heterozygosity or compound heterozygosity. The selected candidate variant was validated in the proband and analyzed in the other family members through Sanger sequencing, using the ABI BigDye Terminator Sequencing Kit (v3.1) (Applied Biosystems, Foster City, CA, United States) and a 3130xl Genetic Analyzer (Applied Biosystems, Foster City, CA, United States). Sequence electropherograms were analyzed using Chromas LITE (v2.01; Technelysium Pty Ltd., Brisbane, Australia).

## Results

SNP-array analysis did not disclose any non-polymorphic CNV, whereas identified a list of widespread ROH, with a total percentage of 4.7% calculated on autosome chromosomes and consistent with the degree of parental relationship (Kearney et al., 2011). This analysis extended to the healthy siblings allowed to refine the sole patient’s ROH, likely including the disease-gene. WES resulted in adequate coverage and depth. A total of 48,015 variants were identified and filtered using several criteria, obtaining 9,776 high-quality variants with an effect on the coding and splice site regions; 956 variants with frequency ≤ 1% in gnomAD or with unknown frequency; 670 variants with a CADD score ≥ 10, predicted to have a high functional impact on the protein. Variants prioritized on the patient’s exclusive ROH revealed a homozygous missense variant in *FBN3* (NM_032447:c.5434A>G; NP_115823:p.Ile1812Val; rs115948457) mapping within a 6.6 Mb ROH as the most promising variant, while not likely damaging variants were found hypothesizing different transmission patterns. The *FBN3* variant had a frequency of 0.002596 in dbSNP 151, a total frequency of 0.0007317 (0.007421 in the African population) in gnomAD and a CADD score of 19.82. The DANN score was 0.9957. This variant was reported in the homozygous state only once in gnomAD. It is predicted as “probably damaging” by PolyPhen-2, as “damaging” by FATHMM, FATHMM-MKL, and FATHMM-XF, and as “disease causing” by MutationTaster. According to the American College of Medical Genetics (ACMG) guidelines, this variant was classified as likely benign considering its frequency in Africans (VarSome, https://varsome.com/), although it is much rarer in the other populations, or as a variant of uncertain significance (VUS) (InterVar, https://wintervar.wglab.org/; Franklin, https://franklin.genoox.com/). Sanger sequencing confirmed the presence of the homozygous variant in the proband (II:2); both the parents (I:1, I:2), one sister (II:3) and the youngest brother (II:4), all non-affected, resulted heterozygous, while the other non-affected sister (II:1) homozygous wild-type, corroborating that the variant co-segregated with the disease. Two missense variants in the same gene, NM_032447:c.3616G>A; NP_115823:p.Val1206Ile; rs747241812 and NM_032447:c.6037C>T; NP_115823:p.Arg2013Trp; rs183278638, were described in compound heterozygosity in two Chinese siblings with clinical diagnosis of BBS and showed developmental delay/cognitive impairment and speech delay, obesity, diabetes mellitus, dental anomalies, and retinitis pigmentosa. Comparison of those clinical features with the clinical characteristics of the patient herein reported showed high similarity, supporting the pathogenicity of the homozygous missense variant detected (Table 1).

**TABLE 1:** Left: diagnostic features of Bardet–Biedl syndrome with their relative prevalence (Forsyth and Gunay-Aygun, 2003; Forsythe and Beales, 2013) and (center) clinical characteristics of our (II:2) and Wang’s patients (III:1 and III:2; Wang et al., 2017). Four primary features or three primary features and two secondary features establish the diagnosis of Bardet–Biedl syndrome (Forsyth and Gunay-Aygun, 2003). Right: clinical characteristics (in bold those showed by all reported patients) of the BBS-like phenotype herein described. N: absent; n.r.: not reported; Y: present.

Diagnostic features of Bardet–Biedl syndrome			II:2	III:1 Wang et al., 2017	III:2 Wang et al., 2017	Clinical characteristics of *FBN3*-related phenotype
Major features	Retinal dystrophies (94%)		N	Y	Y	
	Postaxial polydactyly (79%)		N	N	N	
	Central obesity (89%)		Y	Y	Y	**Obesity**
	Hypogonadism and genitourinary abnormalities (59%)		Y	Y	Y	**Genital anomalies**
	Kidney disease (52%)		N	N	N	
	Cognitive impairment (66%)		Y	Y	Y	**Cognitive impairment**
Minor features	Neurologic abnormalities	Behaviour/psychiatric abnormalities (35%)	N	n.r.	n.r.	
		Speech abnormalities (54–81%)	Y	Y	Y	**Speech abnormalities**
		Epilepsy (9.6%)	N	n.r.	n.r.	
		Developmental delay (81%)	Y	Y	Y	**Developmental delay**
		Ataxia/poor coordination	N	n.r.	n.r.	
	Diabetes mellitus (15.8%)		N	N	Y	Diabetes mellitus
	Oral anomalies (50%)	Dental anomalies	Y	Y	Y	**Dental anomalies**
		Oral abnormalities	Y	n.r.	n.r.	Arched palate
	Cardiovascular and other thoraco-abdominal abnormalities (1.6–29%)		N	Y	N	Cardiovascular defects
	Gastrointestinal abnormalities		N	n.r.	n.r.	
	Olfactory dysfunction (anosmia/hyposmia) (47–100%)		N	n.r.	n.r.	
	Brachydactyly/syndactyly		Y	N	N	Brachydactyly/syndactyly
	Other eye abnormalities	Strabismus	Y	N	N	Strabismus

## Discussion

Autozygosity, or the inheritance of two copies of an ancestral allele, facilitates the mapping of mutations by flagging the surrounding haplotypes as tractable runs of homozygosity, a process known as autozygosity mapping (Alkuraya, 2013). This approach has been successfully used to discover new disease-genes in consanguineous families (Vahidnezhad et al., 2018) and the application of SNP-array and WES analyses together with advanced bioinformatic filtering approaches, focused on likely damaging homozygous variants, accelerated and made more cost-effective this process with an exponential increase of diagnostic yield (Dharmadhikari et al., 2019). We reported a north-African patient born from two first cousins with a novel homozygous variant of *FBN3*, the less characterized member of fibrillin protein family, detected by an integrated analysis of familial SNP-array and proband’s WES, by prioritizing candidate variants within the sole patient’s ROH. Compound heterozygous variants of *FBN3* have been recently described in two siblings with a clinical diagnosis of Bardet–Biedl syndrome (BBS) (Wang et al., 2017). BBS is a rare autosomal recessive syndrome (with an incidence of 1/160,000 and 1/13,500 in some Arab populations), characterized by major symptoms with very variable penetrance, such as retinal dystrophy, obesity, post-axial polydactyly, renal dysfunction, learning difficulties, hypogonadism, and other minor clinical characteristics (Forsythe and Beales, 2013; Niederlova et al., 2019; Forsyth and Gunay-Aygun, 2003). Seen the extremely variable clinical spectrum, four primary features or three primary features and two secondary features are necessary to establish the diagnosis (Forsyth and Gunay-Aygun, 2003) reported in Table 1. The two previously reported patients presented both with intellectual disability, developmental delay, learning disorders, obesity, and genital anomalies, associated with ocular defects, such as retinitis pigmentosa, nystagmus and blepharophimosis, diabetes mellitus, and dental anomalies, all criteria sufficient to diagnose BBS (Wang et al., 2017). Interestingly, also the patient herein reported fulfills the diagnostic criteria of BBS, having three primary (obesity, genital anomalies, and cognitive impairment) and three secondary BBS features (developmental and speech delay, oral/dental anomalies, and brachydactyly/syndactyly). It is noteworthy that in all these cases the polydactyly, one of the most common clinical characteristics of the syndrome, is absent.

BBS is caused by mutations in numerous BBS genes, all encoding proteins involved in cilia biogenesis and function, and is consequently considered a multisystemic ciliopathy (Forsythe and Beales, 2013). Most of these genes encode for the subunits of the BBSome, an octameric protein complex; while others encode for chaperonin assisting the BBSome assembly. Last, the third most common group of genes encodes a GTPase assisting the BBSome function (Forsythe and Beales, 2013). Therefore, BBS is considered a disease resulting from dysfunction of primary cilia, involved in cellular trafficking and transport, although the pathogenesis of the majority of BBS clinical defects has yet to be elucidated.

Overall, pathogenic variants detected in BBS genes account for about 80% of BBS patients (Forsythe and Beales, 2013), suggesting that other genes not yet discovered are responsible for a subset of cases.

The variant herein reported is suggested to cause a distinct phenotype milder than BBS with an estimated frequency, calculated based on its frequency reported in the international database, of 1/20,000 in the African population and of 1/1,800,000 in general.

**FIGURE 2:**
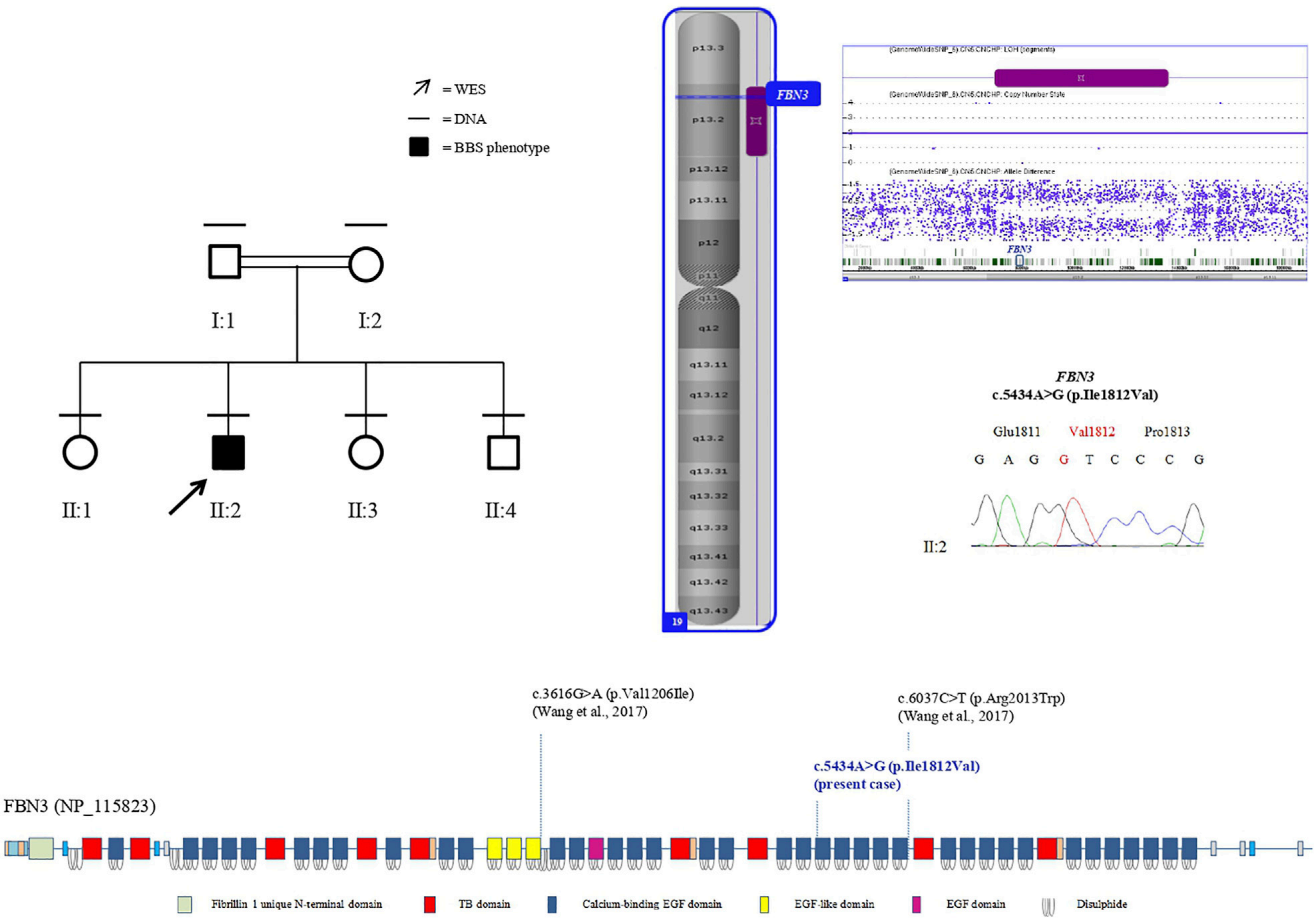
Left: pedigree of the family. Black lines indicate individuals for whom DNA was collected; the arrow indicates the proband that underwent WES. Right: the sole patient’s ROH (purple bar) on chromosome 19, including *FBN3*, assessed by SNP-array analysis (imaged by ChAS software, v3.1). Electropherogram shows the proband’s homozygous genotype of *FBN3* (c.5434A>G) variant. Below: protein domains scheme of FBN3 reconstructed from Pfam (https://pfam.xfam.org/protein/Q75N90) and localization of variants reported so far.

*FBN3*, also known as *KIAA1776*, encodes the less characterized member of the human fibrillin family. The *FBN3* gene has 64 exons and maps on chromosome 19p13.2. The protein is composed of 2,809 amino acids and shows a N-terminal signal sequence, numerous epidermal calcium-binding growth factor (EGF) domains, and some EGF or EGF-like domains, all including six cysteine residues, and several TGF β-binding protein-like (TB) domains, made of eight cysteine modules (Hubmacher et al., 2006; Piha-Gossack et al., 2012) (Figure 2). The C-terminal half of FBN3 multimerizes and strongly interacts with fibronectin, similar to the C-terminal halves of FBN1 and FBN2 (Sabatier et al., 2009).

An EGF domain contains six characteristic cysteine residues, which form three disulfide bonds in a 1–3, 2–4, and 5–6 arrangement, and frequently a consensus sequence for calcium-binding at its N-terminus, representing an essential structural and functional domain of the protein and conceivably mediating protein–protein interactions (Piha-Gossack et al., 2012). The missense variant p.Ile1812Val herein identified falls in a conserved amino acid within one of the calcium-binding EGF domains (https://pfam.xfam.org/protein/Q75N90) and a difference in the properties between the wild-type isoleucine and the mutant valine might disturb the EGF domain structure. It is not possible to assess the functional consequences of this variant on the protein. Anyway, it is noteworthy that the reciprocal substitution, a valine replaced by an isoleucine, in a EGF-like domain has been detected in the previously reported patients (Wang et al., 2017) (Figure 2) and the same amino acid change, an isoleucine replaced by a valine, has been also described in *FBN1* in two patients affected by *FBN1*-related disease: p.Ile724Val (Tjeldhorn et al., 2006), and p.Ile2616Val (Howarth et al., 2007), both falling in a calcium-binding EGF-like domain and classified as VUS and likely pathogenic, respectively, following the most recent classification criteria (https://franklin.genoox.com/).

*FBN3* is highly expressed in numerous tissues in the early stages of embryo development, from the 6th to the 12th gestational week: perichondrium, perineurium, perimysium, skin, developing bronchi, glomeruli, pancreas, kidney, heart, and testis and at the prospective basement membranes in developing epithelia and endothelia (Corson et al., 2004; Sabatier et al., 2011).

FBN3 is also found in the connective tissue of both the central nervous system and the peripheral nervous system (Sabatier et al., 2011). Moreover, expression studies detected high levels of *FBN3* in the developing brain (Corson et al., 2004), but also in the adult brain (Nagase et al., 2000) and this expression pattern distinguishes *FBN3* from the other fibrillins.

How deleterious variants in *FBN3* could lead to a BBS phenotype has yet to be clarified. Seen the high rate of homology between fibrillins and taking into account that *FBN3* is globally expressed in the same organs of *FBN1* and *FBN2*, with some differences suggestive of FBN3 unique functions in certain tissues (Corson et al., 2004; Sabatier et al., 2011), insights inferred from the knowledge of other fibrillin family members can be used to deduct the possible clinical consequences of FBN3 impairment.

Fibrillins play both a well-known structural and an instructive role reflecting their ability to sequester TGF-β and BMP complexes in the extracellular matrix. In particular, the fibrillin-rich microfibrils concentrate the ligands locally and regulate cell differentiation within a spatial context during organ formation (positive regulation) and restrict their bioavailability and modulate cell performance in a timely fashion during tissue remodeling/repair (negative regulation). Therefore, fibrillins are integral components of a broader biological network orchestrating morphogenetic and homeostatic programs in multiple organ systems (Ramirez and Sakai, 2010).

Known mutations in the fibrillin genes cause a spectrum of phenotypes reflecting their role in the cell, and ranging from the Marfan syndrome to patients displaying only tall stature and mild marfanoid skeletal features (Loeys et al., 2003). Moreover, it is noteworthy that mice lacking *Fbn2* display a limb-patterning defect (syndactyly), due to the impaired balance between chondrogenic outgrowth and interdigital cell death, which are under the control of several signaling molecules, including BMPs (Arteaga-Solis et al., 2001). Interestingly, syndactyly is one of the digit anomalies shown by our patients together with brachydactyly, which is considered another digit defect due to perturbation of BMP trafficking (Mundlos, 2009).

A characteristic of *FBN1*-related diseases is the alteration of subcutaneous adipose tissue (Muthu and Reinhardt, 2020). There is much evidence that abnormal adipose tissue metabolism strongly correlates with elevated TGF-β levels in humans and mouse (Samad et al., 1997; Fain et al., 2005; Yadav et al., 2011). It has also been demonstrated that *FBN1* pathogenic variants lead to an upregulation of TGF-β signaling (Andelfinger et al., 2016; Lin et al., 2020). All these data support the hypothesis that obesity, recorded in all the patients herein described, is caused by an increased TGF-β signaling determined by damaging *FBN3* variants alternating an EGF-like domain.

Overall, type I fibrillinopathies are characterized by clinical symptoms in skeletal, cardiovascular, ocular, and adipose tissues, showing a close overlap with clinical features recorded in the *FBN3*-mutated subjects.

*FBN3* variants could be, therefore, associated with a distinct BBS phenotype and partially overlapping with fibrillin-related disorders and characterized by developmental delay and cognitive impairment, obesity, dental, and genital anomalies, in association with other more variable clinical abnormalities including ocular defects, abnormal development of the extremities, cardiovascular, and palate anomalies (Table 1). The *FBN3* expression pattern in the developing and adult central nervous system could explain the neurodevelopmental disorder shown by the current and previous cases, a clinical feature that is vice versa absent in the other fibrillin-related diseases. Conversely, the absence in all cases described so far of polydactyly, one of the most common clinical characteristics of BBS, could be due to the distinct molecular and cellular pathways altered in *FBN3*-mutated and BBS patients.

In conclusion this is the second study associating *FBN3* with a BBS phenotype and it supports *FBN3* as a candidate gene for a BBS-like syndrome.

Up to date, there is no evidence that FBN3 and BBS proteins are structurally or functionally related and further studies are necessary to investigate the molecular basis of this new clinical condition.

## Data Availability

The datasets for this article are not publicly available due to concerns regarding participant/patient anonymity. Requests to access the datasets should be directed to the corresponding author.

## References

[B1] Alkuraya F. S. (2013). The Application of Next-Generation Sequencing in the Autozygosity Mapping of Human Recessive Diseases. Hum. Genet. 132, 1197–1211. 10.1007/s00439-013-1344-x 23907654

[B2] Andelfinger G. Loeys B. Dietz H. (2016). A Decade of Discovery in the Genetic Understanding of Thoracic Aortic Disease. Can. J. Cardiol. 32, 13–25. 10.1016/j.cjca.2015.10.017 26724507

[B3] Arteaga-Solis E. Gayraud B. Lee S. Y. Shum L. Sakai L. Ramirez F. (2001). Regulation of Limb Patterning by Extracellular Microfibrils. J. Cell Biol. 154, 275–282. 10.1083/jcb.200105046 11470817 PMC2150751

[B4] Corson G. M. Charbonneau N. L. Keene D. R. Sakai L. Y. (2004). Differential Expression of Fibrillin-3 Adds to Microfibril Variety in Human and Avian, but Not Rodent, Connective Tissues. Genomics 83, 461–472. 10.1016/j.ygeno.2003.08.023 14962672

[B5] Dharmadhikari A. V. Ghosh R. Yuan B. Liu P. Dai H. Al Masri S. (2019). Copy Number Variant and Runs of Homozygosity Detection by Microarrays Enabled More Precise Molecular Diagnoses in 11,020 Clinical Exome Cases. Genome Med. 11, 30. 10.1186/s13073-019-0639-5 31101064 PMC6525387

[B6] Ewens K. G. Stewart D. R. Ankener W. Urbanek M. McAllister J. M. Chen C. (2010). Family-based Analysis of Candidate Genes for Polycystic Ovary Syndrome. J. Clin. Endocrinol. Metab. 95, 2306–2315. 10.1210/jc.2009-2703 20200332 PMC2869537

[B7] Fain J. N. Tichansky D. S. Madan A. K. (2005). Transforming Growth Factor β1 Release by Human Adipose Tissue Is Enhanced in Obesity. Metabolism 54, 1546–1551. 10.1016/j.metabol.2005.05.024 16253647

[B8] Forsyth R. Gunay-Aygun M. (2003). “Bardet-Biedl Syndrome Overview,” in GeneReviews® [Internet]. Editors Adam M. P. Ardinger H. H. Pagon R. A. Wallace S. E. Bean L. J. H. Mirzaa G. (Seattle (WA): University of Washington, Seattle). Available from: https://www.ncbi.nlm.nih.gov/books/NBK1363/ (updated 2020 Jul 23).

[B9] Forsythe E. Beales P. L. (2013). Bardet-Biedl Syndrome. Eur. J. Hum. Genet. 21, 8–13. 10.1038/ejhg.2012.115 22713813 PMC3522196

[B10] Howarth R. Yearwood C. Harvey J. F. (2007). Application of dHPLC for Mutation Detection of the Fibrillin-1 Gene for the Diagnosis of Marfan Syndrome in a National Health Service Laboratory. Genet. Test. 11 (2), 146–152. 10.1089/gte.2006.0514 17627385

[B11] Hubmacher D. Tiedemann K. Reinhardt D. P. (2006). Fibrillins: from Biogenesis of Microfibrils to Signaling Functions. Curr. Top. Dev. Biol. 75, 93–123. 10.1016/S0070-2153(06)75004-9 16984811

[B12] Jordan C. D. Bohling S. D. Charbonneau N. L. Sakai L. Y. (2010). Fibrillins in Adult Human Ovary and Polycystic Ovary Syndrome: Is Fibrillin-3 Affected in PCOS? J. Histochem Cytochem. 58, 903–915. 10.1369/jhc.2010.956615 20855553 PMC2942743

[B13] Kearney H. M. Kearney J. B. Conlin L. K. (2011). Diagnostic Implications of Excessive Homozygosity Detected by SNP-Based Microarrays: Consanguinity, Uniparental Disomy, and Recessive Single-Gene Mutations. Clin. Laboratory Med. 31, 595–613. 10.1016/j.cll.2011.08.003 22118739

[B14] Kircher M. Witten D. M. Jain P. O'Roak B. J. Cooper G. M. Shendure J. (2014). A General Framework for Estimating the Relative Pathogenicity of Human Genetic Variants. Nat. Genet. 46, 310–315. 10.1038/ng.2892 24487276 PMC3992975

[B15] Lin M. Liu Z. Liu G. Zhao S. Li C. Chen W. (2020). Deciphering Disorders Involving Scoliosis and COmorbidities (DISCO) studyGenetic and Molecular Mechanism for Distinct Clinical Phenotypes Conveyed by Allelic Truncating Mutations Implicated in FBN1. Mol. Genet. Genomic Med. 8, e1023. 10.1002/mgg3.1023 31774634 PMC6978264

[B16] Liu H.-Y. Zhou L. Zheng M.-Y. Huang J. Wan S. Zhu A. (2019). Diagnostic and Clinical Utility of Whole Genome Sequencing in a Cohort of Undiagnosed Chinese Families with Rare Diseases. Sci. Rep. 9 (1), 19365. 10.1038/s41598-019-55832-1 31852928 PMC6920370

[B17] Liu X. Wu C. Li C. Boerwinkle E. (2016). dbNSFP v3.0: A One-Stop Database of Functional Predictions and Annotations for Human Nonsynonymous and Splice-Site SNVs. Hum. Mutat. 37, 235–241. 10.1002/humu.22932 26555599 PMC4752381

[B18] Loeys B. L. Matthys D. M. de Paepe A. M. (2003). Genetic Fibrillinopathies : New Insights in Molecular Diagnosis and Clinical Management. Acta Clin. Belg. 58, 3–11. 10.1179/acb.2003.58.1.001 12723256

[B19] Mundlos S. (2009). The Brachydactylies: a Molecular Disease Family. Clin. Genet. 76, 123–136. 10.1111/j.1399-0004.2009.01238.x 19790289

[B20] Muthu M. L. Reinhardt D. P. (2020). Fibrillin-1 and Fibrillin-1-Derived Asprosin in Adipose Tissue Function and Metabolic Disorders. J. Cell Commun. Signal. 14, 159–173. 10.1007/s12079-020-00566-3 32279186 PMC7272526

[B21] Nagase T. Kikuno R. Hattori A. Kondo Y. Okumura K. Ohara O. (2000). Prediction of the Coding Sequences of Unidentified Human Genes. XIX. The Complete Sequences of 100 New cDNA Clones from Brain Which Code for Large Proteins *In Vitro* . DNA Res. 7 (6), 347–355. 10.1093/dnares/7.6.347 11214970

[B22] Niederlova V. Modrak M. Tsyklauri O. Huranova M. Stepanek O. (2019). Meta‐analysis of Genotype‐phenotype Associations in Bardet‐Biedl Syndrome Uncovers Differences Among Causative Genes. Hum. Mutat. 40, 2068–2087. 10.1002/humu.23862 31283077

[B23] Piha-Gossack A. Sossin W. Reinhardt D. P. (2012). The Evolution of Extracellular Fibrillins and Their Functional Domains. PLoS One 7, e33560. 10.1371/journal.pone.0033560 22438950 PMC3306419

[B24] Ramirez F. Sakai L. Y. (2010). Biogenesis and Function of Fibrillin Assemblies. Cell Tissue Res. 339, 71–82. 10.1007/s00441-009-0822-x 19513754 PMC2819175

[B25] Reinhardt D. P. (2014). Microfibril-associated Disorders. J. Glaucoma 23 (8 Suppl. 1), S34–S35. 10.1097/IJG.0000000000000114 25275902

[B26] Riggs E. R. Andersen E. F. Cherry A. M. Kantarci S. Kearney H. Patel A. (2020). Technical Standards for the Interpretation and Reporting of Constitutional Copy-Number Variants: a Joint Consensus Recommendation of the American College of Medical Genetics and Genomics (ACMG) and the Clinical Genome Resource (ClinGen). Genet. Med. 22 (2), 245–257. 10.1038/s41436-019-0686-8 31690835 PMC7313390

[B27] Robinson P. N. Arteaga-Solis E. Baldock C. Collod-Beroud G. Booms P. De Paepe A. (2006). The Molecular Genetics of Marfan Syndrome and Related Disorders. J. Med. Genet. 43, 769–787. 10.1136/jmg.2005.039669 16571647 PMC2563177

[B28] Sabatier L. Chen D. Fagotto-Kaufmann C. Hubmacher D. McKee M. D. Annis D. S. (2009). Fibrillin Assembly Requires Fibronectin. MBoC 20, 846–858. 10.1091/mbc.e08-08-0830 19037100 PMC2633374

[B29] Sabatier L. Miosge N. Hubmacher D. Lin G. Davis E. C. Reinhardt D. P. (2011). Fibrillin-3 Expression in Human Development. Matrix Biol. 30, 43–52. 10.1016/j.matbio.2010.10.003 20970500 PMC4961473

[B30] Samad F. Yamamoto K. Pandey M. Loskutoff D. J. (1997). Elevated Expression of Transforming Growth Factor-β in Adipose Tissue from Obese Mice. Mol. Med. 3, 37–48. 10.1007/bf03401666 9132278 PMC2230108

[B31] Stewart D. R. Dombroski B. A. Urbanek M. Ankener W. Ewens K. G. Wood J. R. (2006). Fine Mapping of Genetic Susceptibility to Polycystic Ovary Syndrome on Chromosome 19p13.2 and Tests for Regulatory Activity. J. Clin. Endocrinol. Metab. 91, 4112–4117. 10.1210/jc.2006-0951 16868051

[B32] Tjeldhorn L. Rand-Hendriksen S. Gervin K. Brandal K. Inderhaug E. Geiran O. (2006). Rapid and Efficient FBN1 Mutation Detection Using Automated Sample Preparation and Direct Sequencing as the Primary Strategy. Genet. Test. 10 (4), 258–264. 10.1089/gte.2006.258-264 17253931

[B33] Urbanek M. Woodroffe A. Ewens K. G. Diamanti-Kandarakis E. Legro R. S. Strauss J. F. 3rd (2005). Candidate Gene Region for Polycystic Ovary Syndrome on Chromosome 19p13.2. J. Clin. Endocrinol. Metabolism 90, 6623–6629. 10.1210/jc.2005-0622 16091490

[B34] Vahidnezhad H. Youssefian L. Jazayeri A. Uitto J. (2018). Research Techniques Made Simple: Genome-wide Homozygosity/Autozygosity Mapping Is a Powerful Tool for Identifying Candidate Genes in Autosomal Recessive Genetic Diseases. J. Investigative Dermatology 138, 1893–1900. 10.1016/j.jid.2018.06.170 30143075

[B35] Wang K. Li M. Hakonarson H. (2010). ANNOVAR: Functional Annotation of Genetic Variants from High-Throughput Sequencing Data. Nucleic Acids Res. 38, e164. 10.1093/nar/gkq603 20601685 PMC2938201

[B36] Wang Y. Garraoui A. Zeng L. Lai M. He F. Wang H. (2017). FBN3 Gene Involved in Pathogenesis of a Chinese Family with Bardet-Biedl Syndrome. Oncotarget 8, 86718–86725. 10.18632/oncotarget.21415 29156830 PMC5689720

[B37] Yadav H. Quijano C. Kamaraju A. K. Gavrilova O. Malek R. Chen W. (2011). Protection from Obesity and Diabetes by Blockade of TGF-β/Smad3 Signaling. Cell Metab. 14, 67–79. 10.1016/j.cmet.2011.04.013 21723505 PMC3169298

